# Hyperbaric Oxygen Therapy and Tissue Regeneration: A Literature Survey

**DOI:** 10.3390/biomedicines10123145

**Published:** 2022-12-06

**Authors:** J. Lindenmann, L. Kamolz, W. Graier, J. Smolle, F.-M. Smolle-Juettner

**Affiliations:** 1 Division of Thoracic and Hyperbaric Surgery, Medical University of Graz, A-8036 Graz, Austria; 2 Division of Plastic, Aesthetic and Reconstructive Surgery, Medical University of Graz, A-8036 Graz, Austria; 3 Gottfried Schatz Research Center for Cell Signaling, Metabolism and Aging, Molecular Biology and Biochemistry, A-8036 Graz, Austria; 4 Institute of Informatics, Statistics and Documentation, Medical University of Graz, A-8036 Graz, Austria

**Keywords:** tissue regeneration, hyperbaric oxygen, mechanism

## Abstract

By addressing the mechanisms involved in transcription, signaling, stress reaction, apoptosis and cell-death, cellular structure and cell-to-cell contacts, adhesion, migration as well as inflammation; HBO upregulates processes involved in repair while mechanisms perpetuating tissue damage are downregulated. Many experimental and clinical studies, respectively, cover wound healing, regeneration of neural tissue, of bone and cartilage, muscle, and cardiac tissue as well as intestinal barrier function. Following acute injury or in chronic healing problems HBO modulates proteins or molecules involved in inflammation, apoptosis, cell growth, neuro- and angiogenesis, scaffolding, perfusion, vascularization, and stem-cell mobilization, initiating repair by a variety of mechanisms, some of them based on the modulation of micro-RNAs. HBO affects the oxidative stress response via nuclear factor erythroid 2-related factor 2 (Nrf2) or c-Jun N-terminal peptide and downregulates inflammation by the modulation of high-mobility group protein B1 (HMGB-1), toll-like receptor 4 and 2 (TLR-4, TLR-2), nuclear factor kappa-B (NFκB), hypoxia-inducible factor (HIF-1α) and nitric oxide (NO•). HBO enhances stem-cell homeostasis via Wnt glycoproteins and mammalian target of rapamycin (mTOR) and improves cell repair, growth, and differentiation via the two latter but also by modulation of extracellular-signal regulated kinases (ERK) and the phosphatidylinositol-3-kinase (PI3K)/protein kinase B (AKT) pathway. The HBO-induced downregulation of matrix metalloproteinases-2 and 9 (MMP-2/-9), rho-associated protein kinase (ROCK) and integrins improve healing by tissue remodeling. Interestingly, the action of HBO on single effector proteins or molecules may involve both up- or downregulation, respectively, depending on their initial level. This probably mirrors a generally stabilizing potential of HBO that tends to restore the physiological balance rather than enhancing or counteracting single mechanisms.

## 1. Introduction

Hyperbaric Oxygenation (HBO) defines therapeutic hyperoxygenation that has been used in clinical routine for more than six decades. HBO involves treatment in hyperbaric chambers where patients breathe 100% oxygen under elevated ambient pressure. In direct correlation with the pressure level, oxygen dissolves in the plasma resulting in an increase in arterial pO_2_. At pressures between 2.0 and 3.0 atmospheres, which are commonly used for therapy, paO_2_ between 1200 and 2000 (mm Hg) is achieved. This considerable dimension of plasma-mediated hyperoxygenation, which even obviates the presence of erythrocytes at three atmospheres, brings about pharmacological effects on the molecular level, that trigger functional and structural changes [1].

Indications for HBO comprise a wide range of diseases including intoxications, severe necrotizing infections, ischemia-reperfusion injury, crush-syndrome, central nervous injury, radiation-induced tissue damage, burn injury and chronic wounds. The common denominators of the therapeutic action of HBO are the modulation of tissue injury and tissue regeneration [2].

Though slightly different according to the various types of tissue, regeneration relies on complex mechanisms that are regulated by injury-responsive genes [3], and the mobilization of progenitor cells which irreversibly replace damaged cells in the scaffolding, differentiate into mature cells and proliferate accordingly [4]. These reactions are basically caused by the signaling of dying cells that triggers an immune response and inflammation [5] while cells surviving the injury respond to the resulting changes in the microenvironment [4].

## 2. Hyperbaric Oxygen and Regeneration

The effects of HBO on tissue regeneration have been studied extensively, both in experimental and clinical settings. By addressing the mechanisms involved in transcription, signaling, stress reaction, apoptosis and cell-death, cellular structure and cell-to-cell contacts, adhesion, migration as well as inflammation, HBO upregulates processes involved in repair while mechanisms perpetuating tissue damage are downregulated [1]. Though a large variety of HBO-induced changes on the molecular level have been described, there are still unresolved aspects in the mechanisms of HBO-induced tissue repair, some of them with specific impacts for well-determined types of tissues or injuries.

In a literature survey covering the last 10 years, we focused on original papers covering well-documented tissue repair mechanisms triggered by HBO. Use of the terms “hyperbaric, oxygen, tissue, repair” yielded 559 English publications in PUBMED, which were reduced to 522 after excluding papers that merely cited HBO as a treatment possibility without further addressing the topic. Adding the search term “mechanism” reduced the number of articles to 72. Thirty-four out of these publications were reviews, and therefore, excluded as were five articles dealing with the mere technical details of HBO (*N* = 2), retrospective case series without addressing mechanisms of HBO action and one unrelated experimental work. The remaining 33 articles were proven to be valid. By cross-referencing we identified four additional papers dating further back. Following an identical pathway, 26 out of 2195 articles in OVID were eligible. After the removal of 12 duplicates, a total of 39 papers were included.

The most prominent effector proteins and molecules involved in the HBO effects were outlined in detail. For an alphabetic list see Table 1. Table 2 lists the hyperbaric protocols, species and the type of study (experimental or clinical).

### 2.1. The Nuclear Factor Erythroid 2-Related Factor 2 (Nrf2)—Axis

Nuclear factor erythroid 2-related factor 2 (Nrf2), which is activated via the phosphatidylinositol-3-kinase (PI3K)/protein kinase B (AKT) pathway, is a redox-sensitive transcription factor involved in the protection from oxidant stress. Intracellular redox homeostasis is largely controlled by Nrf2, which induces the expression of cytoprotective genes, such as NAD(P)H:quinone dehydrogenase 1 (NQO1), superoxide dismutase 1 (SOD1, Cu/Zn-SOD), glutathione peroxidase (GSH-Px), glutathione reductase (GSH-R), glutathione S-transferase (GSH-T), heme oxygenase-1 (HO-1), glucose-6-phosphate dehydrogenase (G6PD), glutamate-cysteine ligase catalytic subunit (GCLc), glutamate-cysteine ligase modifier subunit (GCLM), sulfotransferases (SULFs), thioredoxin reductase (TR) or UDP-glucose dehydrogenase (UGDH) [6]. In addition, Nrf2 fosters the antioxidative potential of the cell [7]. It has been shown that the stimulation of the PI3K/AKT/Nrf2 signaling axis is a central issue in the improvement of the healing process of wounds [8].

HBO causes direct activation of Nrf-2 protein expression in the nucleus [9].

#### Wound Healing

Oxidative stress in wounded tissue induces the release of inflammatory mediators by macrophages and neutrophils resulting in hyperemia and increased permeability which causes edema. The activation of cytokine cascades by oxidative stress may interfere with healing, whilst apoptosis can be induced over various pathways. Nrf-2 can alleviate oxidative stress, thereby enabling wound healing [10].

In a study in patients with diabetic foot ulcers who significantly improved following HBO, Dhamodharan et al. found Nrf2 along with its downstream targets NAD(P) H:quinone oxidoreductase 1 (NQO-1), Heme-oxygenase-1 (HO-1) and Catalase (CAT) significantly increased when compared to controls. Tissue samples revealed higher levels of angiogenesis markers epidermal growth factor (EGF), vascular endothelial growth factor (VEGF), platelet-derived growth factor (PDGF), fibroblast growth factor 2 (FGF-2) and CxC motiv chemokine 10 (interferon gamma induced protein; CXCL10) in the HBO group. Likewise, HBO enhanced endothelial nitric oxide (eNOS) and nitrite concentrations and the levels of neutrophil attractant interleukin-8 (CxC motif chemokine ligand 8; CXCL-8) which promotes the release of monocyte chemotactic protein 1 (CC chemokine ligand 2; CCL2), a mediator of neovascularization [11]. Godman et al. conducted a genome-wide microarray analysis of gene expression on human microvascular endothelial cells (HMEC-1) after HBO. Again, pronounced Nrf-2-mediated oxidative stress response was one of the primary effects of HBO, whereas normobaric 100% oxygen administration did not provoke a comparable effect. The increased expression of cytoprotective genes induced by HBO corresponded with enhanced cell proliferation and resistance to oxidative stress. Early transcription factors (FOS, FOSB, and JUNB) and metallothioneins as well as six molecular chaperones involved in protein damage control were upregulated immediately following HBOT [12].

### 2.2. Nitric Oxide

Besides causing vasodilation, nitric oxide (NO•) is able to modulate inflammation and eradicate bacterial infections [13] and shows anti- as well as proinflammatory properties, the exact underlying mechanisms of which are unknown [14,15]. Rising concentrations of interleukin one (IL-1) and tumor necrosis factor α (TNF-α) activate the inducible nitric oxide synthase (iNOS) resulting in the synthesis and release of NO• which serves as an immune regulator in the wound. Apart from killing pathogens, NO* can combine with free oxygen radicals to form highly toxic peroxynitrite and hydroxyl radicals [16]. The induction of VEGF and basic fibroblast growth factor (bFGF) NO• enhances wound healing, angiogenesis and muscle regeneration [17]. The anti-inflammatory effects of NO* are most likely caused by enhanced T cell apoptosis and by inhibition of cytokine expression. On the other hand, experiments with knockout mice have shown that NO* increases the levels of pro-inflammatory cytokines such as TNF-α, IL-2, IL-1ß or IFN-γ [14].

Via stimulation of nitric oxide synthase (NOS) HBO increases NO• levels in perivascular tissues [18]. In a study, using cell cultures Venetsanou et al. investigated the basic effects of HBO on NO•. The data showed a time-dependent dynamic change for NO• as well as the activation of oxidant-antioxidant mechanisms regardless of cell type. In general, the findings suggested a central role for NO• in HBO treatment effects [19].

#### 2.2.1. Wound Healing

In a clinical study in patients with chronic wounds, Boykin et al. found a significant increase in local wound NO• levels after HBO suggesting that this mechanism may be involved in improving wound healing and wound closure [20]. Gajendrareddy et al. used a stress model in wounded mice to study the gene expression of wound iNOS. Expression of iNOS increased on days 1, 3 and 5 post-wounding by more than 200%, whereas HBO treatment of the stressed animals significantly reduced iNOS expression while improving healing to near-control levels [21]. Kendall et al. exposed endothelial cells to hypoxia in the presence of lipopolysaccharide and TNF-α, mimicking chronic wound conditions. Out of 20 genes involved in adhesion, angiogenesis, inflammation, and oxidative stress 19 were downregulated five hours after HBO. Only angiogenin gene expression promoting both angiogenesis and NO• production, reflected by increased eNOS expression, was upregulated. Accordingly, pro-inflammatory endothelial IL-8 mRNA and protein decreased. The effect subsided after one day, underlining the rationale for daily HBO applications as usually conducted in clinical routine [22]. In another study, using the same chronic wound model the very authors examined endothelial cells deriving from human umbilical veins and neutrophils. Treatment with HBO decreased hydrogen peroxide generation by neutrophils and induced NO-related protein modifications. In addition, it reversed neutrophil adhesion to endothelial cells whilst the expression of endothelial intercellular adhesion molecule-1 (ICAM-1) and vascular cell adhesion molecule-1 (VCAM-1) were reduced [23]. As mentioned above, Dhamodharan et al. also documented a significant post-HBO increase in eNOS and nitrite concentrations in patients with diabetic foot ulcers [11]. Both the hypoxic gradient and NO in the bone marrow are central for the mobilization of endothelial progenitor cells (EPC) which promote wound healing. Goldstein et al. demonstrated that HBO increases bone marrow NO in vivo, subsequently enhancing the release of EPC. Pretreatment with a NOS inhibitor stalled the HBO effect [18].

#### 2.2.2. Muscle Regeneration

Yamamoto et al. were able to show, that HBO promoted early blood vessel formation and muscle healing after experimental contusion in rats, promoting angiogenesis via the induction of NO• production. Accordingly, HBO significantly increased NO_3_^−^, VEGF, and bFGF levels and stabilized HIF1α within 1 day. The administration of a ROS inhibitor N-acetylcysteine (NAC) or the NOS-inhibitor (L-NAME) before HBO, suppressed angiogenesis and muscle regeneration even after HBO [17].

#### 2.2.3. Abrogating Ischemia-Reperfusion Injury

In a rat model, Baynosa et al. investigated the impact of NOS release on the HBO-induced positive effect on ischemia-reperfusion injury. Early-phase NOS activity in pulmonary tissue was significantly increased after HBO compared with untreated controls. After 4 h of ischemia and 24 h of reperfusion eNOS mRNA and pulmonary protein in HBO-treated animals increased significantly. They concluded, that in ischemia-reperfusion injury the eNOS-dependent effects of HBO may result from a systemic early increase in eNOS enzymatic activity followed by a late-phase increase in eNOS protein expression [24].

### 2.3. Hypoxia Inducible Factor 1α ( HIF-1α)

Apart from disturbances in tissue homeostasis, hypoxic conditions at the wound site recruit macrophages and affect their function. Hypoxia-inducible factor (HIF-1α) is a transcription factor for inflammatory genes and plays a central role in macrophage activation. Biological regulation of monocytes/macrophages in response to circulating HIF-1α has essential roles in healing [25]. HIF-1α also regulates the expression of both α and ß integrins and is involved in the expression of heat-shock protein-90, vascular endothelial growth factor, the chemokine stromal cell-derived factor-1 and GLUT-1, a protein facilitating insulin-independent glucose uptake in tissues. Since all these substances are involved in wound healing, the pharmacological stabilization of HIF-1α enhances healing processes [26,27,28].

#### 2.3.1. Wound Healing

In a diabetic mice wound model, Sunkari et al. documented a significant effect of HBO on HIF-1α at several levels, increasing both HIF-1α stability and activity, thereby inducing fibroblast proliferation [29]. In another study in patients with diabetic foot ulcers post-HBO, HIF-1α, nuclear factor Kappa-B (NFκB) and VEGF expression in wounds as well as circulating inflammatory cytokines were determined. All patients experienced relapse-free healing one month after HBO. HIF-1α, NFκB a protein complex involved in DNA transcription, cytokine production and cell survival, and VEGF expression increased alongside with an increase in fibrous tissue and angiogenesis, as did proinflammatory IL-6, while interferon-gamma decreased [30]. Similarly, Huang et al. exposed human skin fibroblast and human umbilical vein endothelial cells to high glucose and hyperbaric oxygen for studying patterns of gene expression and healing capacity in vitro. Alongside improved healing, HBO enhanced the expression of HIF-1α, NFκB, VEGF, CXC motif chemokine 12 (SDF-1), and CXC-motif chemokine receptor 4 (CXCR4) [31]. HBO promoted proliferation, migration, and the production of reactive oxygen species (ROS) [21].

Zhang et al. studied the effect of HBO on HIF-1-α expression by using ischemic skin flaps in a rat model of wound ischemia. Whilst HIF-1-α expression in wound extract increased and peaked on day 7 in the controls, both the increase and peak level were significantly reduced following HBO. The same was true for the expression of the cell cycle regulator p53. Though these findings on HIF-1-α contradict the abovementioned ones, HBO attenuated apoptosis as mirrored by increased expression of the anti-apoptotic key regulator B-cell lymphoma 2 (Bcl-2) while decreasing cleaved caspase-3, a pro-apoptotic mediator. The downregulation of inflammation went along with the reduction in VEGF, and the pro-inflammatory cyclooxygenase-2 (Cox2), and neutrophil infiltration in the ischemic wounds, which showed improved healing following HBO [32].

#### 2.3.2. Regeneration following Stroke

Rats underwent HBO seven days after temporary middle cerebral artery occlusion. The treatment enhanced functional recovery and neurogenesis as mirrored by increased expression of neurogenin-1, synapsin-1 and doublecortin. The therapeutic action was most likely triggered by increasing ROS and HIF-1α since the inhibition of ROS and HIF-1α abrogated the effect of HBO [33]. Hadanny et al. hypothesized that the repeated hyperoxia resulting from the usual HBO treatment schedules induces the regeneration of neuronal tissue by increasing HIF in a similar way as hypoxia, yet without the deleterious effects of the latter [34].

#### 2.3.3. Bone Regeneration

In cell culture and human peripheral blood monocytes, the effect of HBO on osteoclastic activity was studied. HBO significantly decreased the osteoclast formation and bone resorption induced by the Receptor activator of nuclear factor kappa-Β ligand (RANKL), a central regulator of bone metabolism. The suppressive action of HBO was at least in part mediated through inhibition of hypoxia-induced HIF-1α mRNA and protein expression and reduction in Nuclear factor of activated T-cells, cytoplasmic 1 (NFATc1) and dendritic cell-specific transmembrane protein (Dc-STAMP) expression, both of which are involved in osteoclast differentiation and development [35].

### 2.4. Matrix Metalloproteinases (MMP)

Matrix metalloproteinases 2 and 9 (MMP-2/-9), that are in part regulated by levels of IL-1β, are key tissue remodeling enzymes involved in the degradation of extracellular matrix components. They have multiple overlapping activities critical for wound healing, and regeneration of skeletal muscle or neural tissue [36,37,38]. While matrix metalloproteinases (MMPs) play important roles in the pathology of wounds, the active form of MMP-9 seems to be the main factor contributing to the non-healing of diabetic ulcers [39].

#### 2.4.1. Wound Healing

Nguyen et al. found that due to a complex mechanism involving a decrease in ROS, HBO lowered the levels of active MMP-9 in diabetic mice, who accordingly showed accelerated wound healing [40].

#### 2.4.2. Regeneration following Spinal Cord Injury and CO-Intoxication

The expression levels of MMP-2, MMP-9, IL-6, and VEGF as well as spinal cord water content were measured in rats with experimental spinal cord injury. In HBO-treated animals, IL-6, as well as MMP-2 und MMP-9 levels, were downregulated, positively correlating with the spinal cord water content, which was lower after HBO than in the controls, whereas VEGF was upregulated. HBO promoted the recovery of neuronal function [41]. Experimental carbon monoxide (CO) intoxication caused a dramatic increase in MMP-9 and caspase-3, whereas the ratio of anti-apoptotic Bcl-2 to pro-apoptotic Bcl-2-associated X protein (BAX) which determines the survival or death of cells following an apoptotic stimulus prominently decreased. HBO rapidly reduced MMP-9 and caspase-3 and restored the balance of Bcl-2 and BAX, thereby inhibiting apoptosis and cell death and facilitating neuronal recovery [42].

#### 2.4.3. Cardiac Remodeling

HBO therapy mitigated adverse left ventricular remodeling caused by streptozotocin-induced diabetes in rats. Apart from preventing LV concentric remodeling, heterogeneous myocyte hypertrophy and fibrosis HBO increased MMP-2 gene expression. Moreover, it inhibited the induction of Transforming growth factor beta 1 (TGFB-1), a cytokine involved in cell growth, and apoptosis as well as MMP-9 mRNAs. HBO normalized TNF-α and VEGF protein expression and attenuated leucocyte infiltration [43].

### 2.5. Mechanistic/Mammalian Target of Rapamycin (mTOR)

Mammalian target of rapamycin (mTOR) is a key substance in regeneration mechanisms. In detail, mTOR signaling regulates cellular processes such as stem-cell maintenance, growth, proliferation, and differentiation, but also autophagy. Its signaling pathway interacts with Protein Kinase B (Akt) [44].

#### Regeneration following Spinal Cord Injury and Peripheral Nerve Injury

Following experimental spinal cord injury in rats Chen et al. described that HBO significantly promoted hind-limb locomotor recovery, whilst spinal cavitation or atrophy receded on MRI. Histological analysis showed that the changes in spinal cord neural structure in rats were markedly restored by HBO-PC treatment. Injury-induced increased levels of p-m-TOR, inflammatory cytokines and apoptosis in the spinal cord were attenuated. Intrathecal administration of an mTOR agonist reversed the abovementioned effects [45]. Liu et al. investigated a rat spinal nerve ligation model and reported that HBO attenuated neuropathic pain. On the molecular level, HBO inhibited autophagy impairment and blocked the activation of the mTOR pathway. The impact of HBO was enhanced after intrathecal administration of rapamycin, which decreases mTOR. Conversely, chloroquine which induces autophagy abrogated the HBO effect [46].

### 2.6. High-Mobility Group Protein B1/Toll-like Receptor 4/NFĸB Pathway

High-mobility group protein B1 (HMGB-1) is a transcription factor that enhances inflammatory processes and binds to the transmembrane proteins Toll-like receptors 4 and 2 (TLR-4, TLR-2), and activates NFκB. This pathway is essential in the acute inflammatory response and immune homeostasis. Hence, it is considered a possible target for therapeutic interventions. Further activation mechanisms of NFκB involve interleukin 1-beta (IL-1ß), or TNF-α [47].

#### Regeneration following Spinal Cord Injury

The inflammatory response to spinal cord injury causes secondary damage and is a crucial factor in regeneration. In a spinal trauma model in rats, Yang et al. applied HBO and found a significant improvement in locomotor activity compared with controls. Concomitantly, HBO downregulated the post-traumatically increased expression levels of NFĸB and HMGB-1 [48]. Sun et al. were able to demonstrate similar effects on signaling and neurological function recovery in patients with acute spinal cord injury; HBO therapy downregulated HMGB1 and NFĸB expression while motor/pain scores improved significantly when compared with controls [49]. The results were further corroborated by Kang et al. who inflicted spinal cord injury in rats which increased gene expressions of TLR-4, HMGB-1, NFκB and the TLR-4 protein expression. All substances significantly decreased after HBO correlating with significant improvement of the motility scores [50]. Tan et al. reported similar findings. They examined TLR-2 as a mediator of the host-response mechanism, NFκB, IL-1β and TNF-α levels following experimental spinal cord injury in rats. All parameters increased post-injury but were attenuated after HBO treatment. Neurological outcomes and histological findings improved accordingly [51].

### 2.7. Protein Kinase B (Akt), Extracellular-Signal Regulated Kinases (ERK), c-Jun-N-Terminal Kinases (JNK)

Activated by cytokines or pathogens, extracellular-signal regulated kinases (ERK) control cellular growth, proliferation, differentiation, and adhesion. The function of the Protein Kinase B (Akt) pathway is similar and additionally involves the regulation of apoptosis and cell survival. c-Jun-N-terminal Kinases are activated as a stress response and regulate intracellular signaling [52].

#### 2.7.1. Regeneration following Brain Injury

Yang et al. used rats for a traumatic brain injury model including neural stem cell transplantation. HBO accelerated neural stem cell proliferation and their migration into the lesion area and increased the levels of ERK, VEGF, VEGR2, rapidly accelerated fibrosarcoma protein kinase (Raf-1) and MEK 1/2 protein kinase which are part of the ERK signaling pathway. Neurological function in HBO-treated rats improved significantly more than in controls [53].

In a mouse model of traumatic brain injury, He et al. documented an increase in apoptotic neurons and mRNA expression, as well as activated caspase 3 protein. In addition, brain injury caused the lowering of pAkt/Akt, and its downstream products phosphorylated/unphosphorylated glycogen synthase kinase 3β (pGSK3β/GSK3β), and β-catenin, both of which instigate neuronal apoptosis. By upregulating of pAkt/Akt, pGSK3β/GSK3β, and the structural protein beta-catenin, HBO attenuated the apoptotic process [54].

Recently, Xia et al. investigated whether HBO therapy exerts neuroprotective effects in a rat model of traumatic brain injury, which provoked an increase in apoptosis in the injured cortex. They found that phosphorylated extracellular signal-regulated kinase (p-ERK), phospho-c-Jun N-terminal kinase (p-JNK) and p-NFκB, as well as CXCL1 and chemokine (C-X-C motif) receptor (CXCR2), increased after experimental trauma. Inhibitors of ERK, JNK and NFκB attenuated the expression of CXCL1 and CXCR2. The same was true for HBO therapy which down-regulated both the expression of ERK and the formation of p-ERK, p-JNK, p-NFκB, CXCL1, and CXCR2, and reduced nerve cell apoptosis, improved the neurological function of rats following traumatic brain injury, and ultimately alleviated the secondary injury [55].

#### 2.7.2. Bone Regeneration

HBO promoted the proliferation of growth-arrested osteoblasts in vitro after 3 days of treatment. It caused significantly increased mRNA expression of fibroblast growth factor (FGF)-2 as well as enhanced protein expression levels of Akt, p-ERK, NFκB, protein kinase Cα (PKCα), and p-JNK [56].

The effect of HBO on the stress-induced JNK signaling pathway was investigated in rats. The administration of IL-1β induced an inflammatory response in chondrocytes, resulting in enhanced expression of JNK and c-Jun. The administration of a selective JNK inhibitor or HBO treatment likewise lowered the expression of JNK and c-Jun, proving that hyperbaric oxygen can inhibit IL-1β induced inflammatory response in chondrocytes by blocking JNK/c-Jun signaling [57].

### 2.8. Rho-Associated Protein Kinase

Rho-associated protein kinase (ROCK) is the effector of Rho-proteins. They interact with the cytoskeleton, regulating smooth muscle tone, and regulating shape, migration and cell-to-cell adhesion. In particular, Rho-proteins influence the actin cytoskeleton, and thereby, smooth muscle tone regulation and seem to be important for modulating tight-junction protein function [58,59].

#### Restoring Intestinal Barrier Function

The disturbance in the intestinal barrier function, which is often associated with spinal cord injury, was studied in an experimental setting. Following spinal cord injury intestinal mucosal injury score, intestinal permeability, and levels of Rho and ROCK1 were higher, whereas tight junction proteins were significantly lower than in controls. HBO downregulated the expression of Rho and ROCK1, whilst the expression of the junctional proteins Occludin and ZO-1 was elevated yielding decreased intestinal permeability while intestinal mucosal injury declined significantly [59].

### 2.9. Wnt/ß-Catenin Signaling

Wnt glycoproteins initiate a signaling pathway important in embryogenesis. In consequence, some hereditary conditions are associated with mutations in Wnt pathway components. They are also involved in stem-cell homeostasis and cellular repair mechanisms. Mutations in Wnt genes are also found in some malignant—predominantly intestinal—tumors [60].

The Wnt signaling pathway plays a central role in bone metabolism, where Wnt regulates the osteogenic differentiation of stem cells and activates osteoblasts [60,61].

#### Bone Regeneration

In an animal model of bone healing using rabbits, HBO caused upregulation of mRNA and levels of Wnt3a, Wnt protein, β-catenin and Runx 2, a transcription factor that is essential for osteoblast differentiation and chondrocyte maturation. Accordingly, HBO treatment resulted in osteogenic differentiation of mesenchymal stem cells as documented by specific staining methods. On the other hand, glycogen synthase kinase-3 (GSK-3β), the over-expression of which causes loss of bone mass, was downregulated showing a complex effect of hyperbaric oxygen on Wnt secretion, processing and signaling. In addition, HBO increased alkaline phosphatase activity and calcium deposition [62].

### 2.10. Micro-RNA (Non-Coding RNA)

Micro-RNA or non-coding RNA are highly specific regulators of genes at the post-transcriptional level. They effectuate a sort of “fine-tuning” of gene expression by reducing the latter or by silencing genes, either inhibiting translation or causing the degradation of RNA [63].

#### 2.10.1. Neuronal Regeneration (Stroke Model)

Pyroptosis is a type of lytic cell death and is usually found as a reaction to intracellular infection. It is triggered by inflammasomes that contain a set of caspases that eventually cause the eradication of the initial stimulus. Recently it has been shown that oxygen-glucose deprivation following a stroke can provoke pyroptosis of neural stem cells. In a culture of murine neural stem cells, oxygen-glucose deprivation-induced nucleotide-binding oligomerization domain-like receptors (NOD)-like receptor protein 3 (NLRP-3) inflammasome expression caused the pyroptosis of neuronal stem cells. HBO protected neural stem cells against pyroptosis by modulating the complex inflammasome axis (lncRNA-H19/miR-423-5p/NLRP3 axis) resulting in the upregulation of miR-423-5p which inhibited NLRP3 expression. Thereby neurogenesis was enhanced [64].

#### 2.10.2. Regeneration of Cartilage

In degenerated discs, the expression of both high-mobility group box 1 (HMGB1) and receptor for advanced glycation end-products (RAGE) are upregulated as part of the inflammatory reaction. The micro-RNA (miR107) which modulates HMGB1 by a matching sequence is downregulated during hypoxia. HBO markedly induced miR-107, which caused simultaneous suppression of HMGB1 and thereby attenuated the inflammatory reaction. In addition, HBO downregulated the cytokine-induced extracellular signal-regulated protein kinase (ERK), and c-Jun N- terminal kinase (JNK) as well as the secretion of MMP-3, MMP-9, and MMP-13 [65].

Like degenerated discs, osteoarthritic cartilage shows the downregulation of micro-RNA 107 expression, whereas the proinflammatory HMGB-1, Toll-like receptors (TLRs), and RAGE are upregulated. In rabbit cartilage, HBO induced miR-107 expression which caused marked simultaneous suppression of HMGB-1 by downregulation of the mRNA and protein expression of HMGB-1 and the secretion of HMGB-1. Moreover, RAGE, TLR2, TLR-4, and iNOS, NFκB, and the secretion of MMPs were markedly reduced. Accordingly, immunohistochemistry showed enhanced cartilage repair following HBO. The HBO effect could be reversed by the knockdown of miR107 [66].

In a more recent study in intervertebral disc degeneration in human nucleus pulposus cells, Lin et al. investigated the effect of HBO on microRNA 573 (miR-573) which was found downregulated in the degenerative cells, whereas the pro-apoptotic Bcl-2-associated X protein (BAX) levels, the mRNA of which contained a matching sequence for miR-573 were increased. HBO induced MiR-573 resulting in the suppression of BAX in the cartilage cells. Accordingly, the overexpression of miR-573 following HBO inhibited apoptosis and enhanced proliferation. As in the preceding study, transfection with an antagonist of miR-573 partly suppressed the effects of HBO [67].

#### 2.10.3. Angiogenesis

Metastasis-associated lung adenocarcinoma transcript 1 (MALAT1) is a proangiogenic long noncoding RNA. Shyu et al. investigated the effect of HBO-induced MALAT1 exosomes from cardiac myocytes on myocardial infarction in rats. They found that HBO upregulated MALAT1 thereby increasing cell proliferation and capillary-like network formation. MALAT1 small interfering RNA (siRNA) also reduced miR-92a expression which exerts an inhibitory effect on the vasoprotective Krüppel-like factor 2 (KLF2). Consequently, KLF2 expression increased following HBO boosting neoangiogenesis. HBO and HBO-induced exosomes significantly increased cell proliferation and capillary-like network formation [68].

### 2.11. Integrins

Integrins are transmembrane receptors responsible for adhesion to both the extracellular matrix and cell-to-cell. They facilitate rapid responses in hemostasis or immunological challenges and are important for wound healing. Their function in signal transduction contributes to proliferation and cellular homeostasis [69].

#### Wound Healing

In a study using neutrophils from patients with chronic, nonhealing wounds HBO dramatically reduced β2 integrin expression on neutrophils one month beyond the end of actual treatment. Cell adhesion function of both neutrophilic integrins, α4β1 and β2 was reduced by 70% and 67%, respectively, corresponding to both a decrease in neutrophil adhesion to the endothelium and recruitment into the perilesional area. Of note, α4β1 integrin remained sensitive to antagonist inhibition in the presence of fibronectin—a fact that may indicate the possibility of combining HBO with integrin antagonists [70].

## 3. Conclusions

Throughout six decades HBO has been applied in a large variety of acute diseases but also with the purpose of tissue regeneration in chronic disease [71]. Especially in wound healing and in regeneration following acute spinal cord or brain injury, single cases and case-controlled trials have shown remarkable results. However, due to logistic problems in creating viable setups for high-quality randomized clinical studies, undisputable data are still scarce. On the other hand, extensive experimental work within the last 20 years is shedding light on the molecular mechanisms of HBO various aspects of which have been summarized in a number of recent reviews [72,73,74,75,76,77].

**Figure 1:**
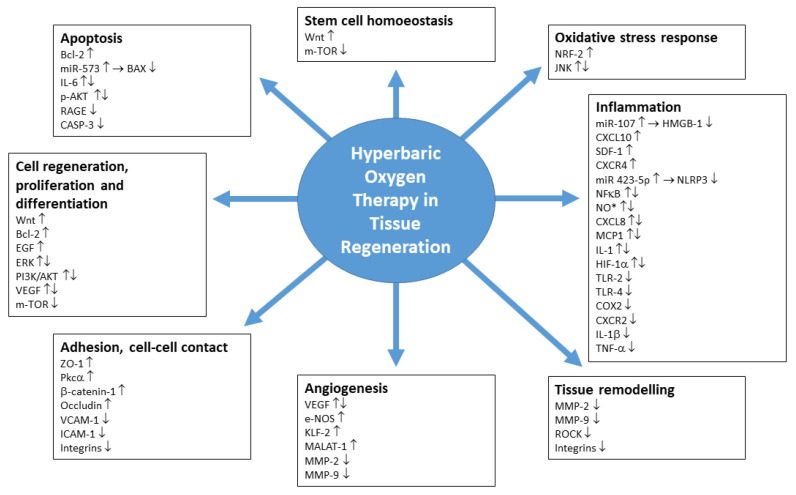
Main points of action of HBO in tissue regeneration.

The present compilation as depicted in Figure 1 puts the focus on conditions where the use of HBO in clinical practice has shown positive effects on regeneration. In particular, these indications involve wound healing, regeneration following brain or spinal cord injury, regeneration of bone and cartilage, cardiovascular issues and muscle regeneration, apart from a multitude of further applications. The list of mechanisms presented herein is far from depicting the complete state of the results.

Most controlled studies have verified that the clinical efficacy of HBO derives from the modulation of signaling and transduction cascades, leading to the synthesis of structural proteins or growth factors and promoting healing and ameliorating post-ischemic and post-inflammatory injuries [78]. Yet, the various substances and pathways that HBO modifies must not be considered separately since many of these very molecular mechanisms are intricately interdependent.

One line of action of HBO is the modulation of ERK and of the PKB/Akt pathway. Both are activated by cytokines or pathogens and control cellular growth, proliferation, differentiation, and adhesion, whereas Akt also involves the regulation of apoptosis and cell survival [52]. One of the targets of Akt is Nrf2, a central component in healing processes. Its upregulation by HBO enhances tissue regeneration in chronic wounds [7,8,11]. Another target of PKB/AKT is mTOR, a pivotal substance regulating regeneration processes. It is involved in stem-cell maintenance, proliferation, and differentiation, growth, cell survival but also autophagy [44]. The downregulation of mTOR by HBO has been connected to the promotion of recovery following experimental spinal-cord injury [79] and alleviation of neuropathic pain [46]. Since Rapamycin potentiates this effect, its combination with HBO is worthwhile to investigate.

Another target of HBO is NO•, the smallest signaling molecule. NO• is a potent vasodilator, enhances synaptic plasticity, modulates inflammatory processes and enhances the release of endothelial progenitor cells. It is produced by three isoforms (iNOS, eeNOS and neuronal nNOS) of synthases [13,80]. Apart from being activated by inflammation mediators such as TNF-α, they respond to HBO as a trigger [11,18,66] which instigates healing processes. What is more, eNOS modulation by HBO seems to be an important issue in abrogating the deleterious effects of ischemia-reperfusion [24]. A seemingly contradictory finding of HBO increasing local NO*-levels in chronic wounds [20] and reducing iNOS expression in wounds under stress conditions [21] might reflect the potential of HBO in restoring homeostasis. Since HBO improved healing in both instances, it may be hypothesized that initial NO- levels had been too low in chronic and too high in stressed wounds, in both cases impeding the healing process. As shown in Table 1 similar, apparently contradictory effects of HBO have been published for other substances.

Chemokines play key roles at each stage of the wound healing process to help coordinate the interaction of cells within the wound. Whilst chemokines act beneficially in early post-wounding, a prolonged inflammatory response exacerbated by chemokines can lead to the formation of chronic non-healing wounds. Therefore, approaches to chemokine inhibition should most likely be adapted to the respective phase the wound is in [81]. HBO seems to fulfill these requirements, as shown by its action on Hypoxia inducible factor and Matrix metalloproteinases. Due to its central role in macrophage activation, Hypoxia inducible factor (HIF-1α) has considerable influence on healing. Macrophages initiate inflammatory immune response following changes in the tissue microenvironment [26,27,28]. In addition, HIF-1α modulates the expression of further substances involved in the healing process such as integrins, heat-shock protein-90, vascular endothelial growth factor, the chemokine stromal cell derived factor-1 and GLUT-1, a protein facilitating insulin-independent glucose uptake in tissues. However, as also found in NO*, the effect of HBO on HIF-1α is not unidirectional. Whereas downregulation was found during the healing of chronic diabetic ulcers [29,31] and a model of bone regeneration [34], HBO instigated upregulation of previously decreased HIF-1α in a model of acute wound ischemia, thereby also improving healing [32]. A similar observation was made in experimental stroke [33]. Thus, as in ERK and Akt, the effect of HBO seems to be a modulating one, ensuring enhanced expression in case of HIF-1α deficiency, whilst downregulating the substance in the presence of chronic inflammation.

The pro-inflammatory IL-1ß which is grossly downregulated by HBO is involved in the regulation of the tissue remodeling enzymes MMP-2 and MMP-9. Metalloproteinases influence not only wound healing but also the regeneration of neural or muscular tissue [34,35,36,37,38]. The multiple and complex functions of MMPs are barely understood, yet the active form of MMP-9 seems to be the main factor contributing to the non-healing of diabetic ulcers [39]. HBO lowered MMP levels in chronic wounds, acute spinal and cerebral injury, and cardiac muscle degradation with beneficial effects [40,41,43]. This is remarkable since MMP inhibitors have not yet yielded convincing therapeutic results [82]. Again, HBO seems to restore physiological balance rather than merely counteracting a substance.

The transcription factor High-mobility group protein B1 (HMGB1) also acts as a cytokine mediator of inflammation. In an essential inflammatory pathway, it binds to the transmembrane proteins TLR-4 and TLR-2, thereby activating NFκB. Alternatively, Interleukin 1-beta (IL-1β) or TNF-α activate NFĸB [46]. Both in clinical and experimental spinal cord injury HBO downregulated HMGB1 and NFĸB as well as TLR, IL-1β and TNF-α levels and yielded morphological and clinical improvement [48,49,50,51].

HBO also influences structural proteins involved in tissue regeneration such as Rho-associated protein kinase (ROCK) which interacts with the cytoskeleton regulating, shape, migration, and cell-to-cell adhesion. In this context HBO downregulated ROCK in spinal cord injury, thereby reducing pathologically increased intestinal permeability [58]. Similarly, the transmembrane receptor family of Integrins regulates intercellular and cellular-extracellular adhesion, thereby also influencing cellular migration as necessary for rapid cellular immune response or hemostasis [69]. HBO-induced downregulation of integrins in neutrophils reduces their adhesion ability, alleviating the inflammatory reaction in chronic wounds [70].

The complex Wnt glycoprotein/β-catenin signaling pathway is involved in embryogenesis and carcinogenesis. In addition, Wnt plays a role in the regeneration of bone, where it regulates the osteogenic differentiation of stem cells. HBO-induced upregulation of Wnt enhanced osteogenic differentiation [60,61,62].

Micro-RNA or non-coding RNA are highly specific regulators of genes at the post-transcriptional level. They effectuate the silencing of genes by either inhibition of translation or by RNA-degradation [63].

In conclusion, though the impact of HBO on various substances seems straightforward, the understanding of its complex effects on transcription, signaling, cell-to-cell interaction and structure is merely scratching the surface. The in-part contradictory action on single effector proteins or molecules which often involves both up-or downregulation, respectively, probably mirrors a generally stabilizing potential of HBO that tends to restore the physiological balance rather than enhancing or counteracting single mechanisms.

**Table 1:** Tissue regeneration. Mechanisms involved in effectiveness of HBO; The arrows indicate the effect of HBO on the level of the effector molecule initially found during respective study (in alphabetical order).

Effector Protein/Molecule	Abbreviation	Field of Action	Change
A-4 integrin [70]	IA4 (CD49D)	Adhesion	↔
A-4beta-1 integrin (very late activation antigen 4) [70]	VLA-4 (CD49DCD29)	Adhesion	↓
Angiogenin (Ribonuclease 5) [22]	ANG (RNASE5)	Transcription	↑
Anti-apoptotic B-cell lymphoma 2 [32,42]	Bcl-2	anti-apoptotic	↑
Bcl-2-associated X protein [42,67]	Bax	pro-apoptotic	↓
Beta catenin-1 [54,62]	CTNNB1	Adhesion; tissue homeostasis	↑
Beta-2 integrin [70]	CD18	Adhesion	↓
C-Jun-N terminal kinases [12,55,56,57,65]	JNK	Stress induced kinases; pro-apoptotic	↑↓
Cleaved caspase 3 [64]	CASP-3	Apoptosis	↓
Cryopyrin [64]	NLRP3 (NACHT)	interacts with components of damaged cells; forms NLRP-inflammasome	↓
Cyclooxygenase 2 (Prostaglandin synthase 2) [32]	COX-2 (PGHS-2)	Pro-inflammatory signaling	↓
CxC motic chemokine Ligand 1 [55]	CXCL1	Inflammatory chemoattractant	↓
CxC motiv chemokine 10 [11] (interferon gamma induced protein)	CXCL10	Inflammation, Signaling	↑
CxC motiv chemokine 12 [31]	SDF-1	Inflammation, Signaling	↑
CxC motiv chemokine ligand 8 [11] (Interleukin 8)	CXCL8 (IL-8)	Transcription, inflammation	↑↓
CXC-Motiv-Chemokinreceptor 2 (IL-8 Receptor beta) [55]	CXCR2	Signaling; pro inflammatory	↓
CXC-motiv-chemokinreceptor 4 [31]	CXCR4	Inflammation, Signaling	↑
Dendritic cell-specific transmembrane protein [35]	Dc-STAMP	co-regulator in osteoclast development	↓
Endothelial nitric oxide gene expression [11,22,24]	eNOS	Signaling (vascular)	↑
Epidermal growth factor [11]	EGF	Induction of mitosis in epithelial cells	↑
Extracellular signal-regulated protein kinase [53,55,56,65]	ERK	Regulates growth and differentiation	↑↓
Fibroblast growth factor 2 (basic fibroblast growth factor 2) [11,17,56]	FGF-2	Transcription	↑
FOS protein [12]	FOS (AP-1; c-fos)	Transcription	↑
FOSB protein [12]	FOSB (G0/G1 switch regula-tory protein 3; G0S3)	Transcription	↑
Glycogen synthase kinase-3 [54,62]	GSK3 beta	proliferation, differentiation, energy metabolism, neuronal development	↑↓
High mobility group box 1 protein [48,65,66]	HMGB1	Cytokine mediator of inflammation	↓
Hypoxia inducible factor 1 α subunit [29,30,31,32,33,35]	HIF-1α (HIF1A)	Regulator of cellular response to oxygen levels	↑↓
Inducible nitric oxide synthase [21,66]	i-NOS (NOS-2)	Signaling	↓↑
Interferon gamma [11,30]	IFG	Cytokine	↓
Interleukin 1beta [51,57]	IL-1 ß (IL-1; IL1B)	Pro-inflammatory cytokine	↓
Interleukin-6 [30,46] (Interferon beta 2)	IL-6 (IFNB2)	apoptosis and proliferation of leucocytes	↑↓
Intracellular adhesion molecule- 1 [23]	ICAM-1 (CD54)	Adhesion	↓
Krüppel-like factor 2 [68]	KLF-2	Vasoprotective; Inhibited by miR-92a	↑
Matrix Metalloproteinase-9 [40,41,42,43,65,66]	MMP-9 (GELB)	Signaling (vascular), migration	↓
Matrix Metalloproteinase-2 [41,43,65,66]	MMP-2 (CLG4A)	tissue remodeling, angiogenesis and cell migration	↓
Matrix Metalloproteinase-13 [65,66]	MMP-13 (CLG3)	Cleaves Type II collagen	↓
Mechanistic target of rapamycin [46,79]	m-TOR (FRAP-1)	Proliferation, differentiation, autophagy	↓
Metastasis associated lung adenocarcinoma transcript 1 [68]	MALAT 1	Transcription	↑
Micro-RNA 92a [68]	miR-92a	Downregulates KLF-2	↓
Monocyte chemotactic protein 1 (CC chemokine ligand 2) [11]	MCP1 (CCL2)	Chemokine secreted by inflammatory cells. Binds to epithelia, attracting and fixating mainly monocytes	↑↓
Nitric oxide (endothelium derived relaxing factor) [17,18,19,20,21,22,23,24,66]	NO (EDRF)	Free radical, causes vasodilation	↑
Nuclear factor erythroid 2-related factor 2 [11,12]	Nrf2	Transcription of cytoprotective genes	↑
Nuclear factor kappa-B [29,31,48,49,50,51,55,56]	NF-ĸB	Transcription, Inflammation	↓↑
Nuclear factor of activated T-cells, cytoplasmic 1 [35]	NFATc1	factor in gene transcription during immune response; involved in regulation of bone-mass	↓
Occludin [59]	OCLN	Tight junctions; preserves barrier function of epithelia	↑
p53 [32]	p53	Transcription	↓
phosphorylated/unphosphorylated glycogen synthase kinase 3 [54,62]	pGSK3beta/GSK3beta	Neuronal Apoptosis	↓
Platelet derived growth factor [11]	PDGF	Transcription	↑
Protein kinase B [54] (phophorylized Akt)	p-Akt	Transcription; regulates proliferation and cell death	↑↓
Protein kinase C A [56]	Pkc α	Adhesion, tight junctions	↑
Receptor for advanced glycation end products [65,66]	RAGE	Induces cell survival, differentiation or apoptosis according to activity	↓
Rho-associtaed protein kinase [59]	ROCK	Regulates cell shape and migration	↓
Runt-related transcription factor 2 [62]	Runx 2	Master regulator of bone development	↑
Toll-like receptor 4 [50,66]	TLR4	pro-inflammatory cytokine signaling	↓
Toll-like receptor 2 [51,66]	TLR2	Pathogen recognition signaling	↓
Transforming growth factor beta 1 [43]	TGFB1	Proliferation, differentiation	↓
Transcription factor JUNB [12]	JUNB	Transcription	↑
Tumor necrosis factor α [22,43,51]	TNF-α	Transcription; pro inflammatory	↓
Reactive oxygen species [31,33,40]	ROS	Signaling; dose dependent effect	↑↓
Vascular cell adhesion molecule-1 [23]	VCAM-1 (CD106)	Adhesion	↓
Vascular endothelial growth factor [11,17,30,31,43,53]	VEGF	Transcription, vascular signaling	↓↑
Wnt family member 3a [62]	Wnt-3a	skeletal differentiation and development; maintenance of bone-mass	↑
Zonula occludens-1 [59]	ZO-1	cross-links and anchors tight junction proteins	↑

**Table 2:** Hyperbaric protocols, type of study.

Ref. No	N HBO Total	N HBO Day	Pressure (bar)	Duration (min)	Species	Tissue/Indication/Experiment
[11]	25	1	2.2	60	Human (patients)	Diabetic foot ulcer
[12]	1	1	2.4	60	Human	Cells (endothelial, microvascular)
[17]	5	1	2.5	120	Rat	Contused muscle
[18]	5–8	1	2.4	90	Mouse	Limb ischemia
[19]	5	1	2.8	60	Human	Cells (fibroblasts, epithelia)
[20]	20	1	2.0	90	Human (patients)	Chronic wound
[21]	5	1	2.5	120	Mouse	Wound model
[22]	1	1	2.4	90	Human	Cells (chronic wound condition)
[23]	1	2	2.4	90	Human	Umbilical cord. neutrophils
[24]	1	1	2.5	90	Rat	Muscle (Reperfusion injury)
[29]	1	1	2.5	60	Human	Cells (monocytes, fibroblasts)
	1	1	2.0	90	Mouse	Diabetic wound model
[30]	20	1	2.4	70	Human (patients)	Diabetic foot ulcer
[31]	30	1	2.0	90	Mouse	Diabetic wound model
[32]	3–14	1	2.4	90	Rat	Ischemic wound model
[33]	7	1	2.5	90	Rat	Stroke model
[35]	4	1	2.4	90	Human	Cells (RAW 264.7, macrophages)
[40]	10	1	2.0	90	Mouse	Wound model
[41]	2–10	2	2.5	60	Rat	Spinal cord injury
[42]	1–21	1	2.5	90	Rat	CO-poisoning
[43]	35	1	2.5	60	Rat	Diabetic heart model
[46]	5	1	2.0	60	Rat	Nerve ligation
[48]	2–20	1–2	2.5	70	Rat	Spinal cord injury
[49]	30	1	2.0	115	Human (patients)	Spinal cord injury
[50]	1–14	1	2.0	70	Rat	Spinal cord injury
[51]	1	1	2.4	60	Rat	Spinal cord injury
[53]	7	1	2.0	110	Rat	Traumatic brain injury
[54]	1	1	2.8	90	Mouse	Traumatic brain injury
[55]	3	1	2.0	60	Rat	Traumatic brain injury
[56]	4	1	2.5	80	Mouse	Cells (osteoblast-like)
[57]	1	every 2nd day	0.5	90	Rat	Chondrocytes
[59]	1–14	1	2.0	60	Rat	Spinal cord injury
[62]	1–7	every 3rd day	2.5	90	Mouse	Traumatic brain inury
[64]	1	1	2.0	70	Mouse	Cells (Neurons hippocampus)
[65]	3	every 2nd day	2.5	120	Human	Cells (nucleus pulposus cells)
[66]	3	every 2nd day	2.5	90	Human	Cells (Chondrocytes)
[67]	3	every 2nd day	2.5	120	Human	Cells (degenerated disc cells)
[68]	1–14	1	2.5	60	Rat	Myocardial infarction model
[70]	15	1	2.45	90	Human (patients)	Chronic wound
[79]	10	1	2.0	100	Rat	Spinal cord injury

## Data Availability

The data used in this review are published in the cited references.
